# Green Approaches in High-Performance Liquid Chromatography for Sustainable Food Analysis: Advances, Challenges, and Regulatory Perspectives

**DOI:** 10.3390/molecules30173573

**Published:** 2025-08-31

**Authors:** Eftychia G. Karageorgou, Natasa P. Kalogiouri, Victoria F. Samanidou

**Affiliations:** 1 NBIS P.C. Cargo Inspections and ISO Certification, 54351 Thessaloniki, Greece; ekarageorgou@nbis.gr; 2 Laboratory of Analytical Chemistry, School of Chemistry, Aristotle University of Thessaloniki, 54124 Thessaloniki, Greece; kalogiourin@chem.auth.gr

**Keywords:** green analytical chemistry, sustainable HPLC, food analysis, eco-friendly solvents, miniaturization, green metrics, food safety management

## Abstract

This review provides a comprehensive overview of the recent green innovations in high-performance liquid chromatography (HPLC) for sustainable food analysis. It outlines the principles of green analytical chemistry and examines advances such as eco-friendly solvent systems, miniaturized and energy-efficient instrumentation, and greener sample preparation techniques. Key applications include the analysis of bioactive compounds, detection of contaminants and residues, and support for clean-label and sustainability claims. Furthermore, the review discusses relevant regulatory and certification frameworks, including ISO 14001, ISO 22000, and global food safety initiatives aligned with environmental, social, and governance standards. Persistent challenges, such as cost, limitations in analytical performance, and limited instrument availability, are highlighted, along with the need for reliable metrics to assess the environmental impact and effectiveness of green analytical practices. The review concludes by emphasizing the need for interdisciplinary collaboration among scientists, industry stakeholders, and regulatory bodies to support the wider adoption of sustainable HPLC practices in food laboratories.

## 1. Introduction

As global demand for safer, more sustainable food systems continues to rise, the analytical sciences are increasingly expected to contribute to environmental protection and the efficient use of resources. Growing environmental concerns have made sustainability a key objective across many scientific disciplines, including analytical chemistry. Traditional analytical methods play an essential role in ensuring food safety and quality, but they often involve the use of hazardous or even toxic solvents, generate large amounts of chemical waste, and require high energy consumption. These drawbacks contribute to environmental pollution and pose occupational health risks, especially in laboratories with high sample processing demands. To address these challenges, the concept of green analytical chemistry (GAC) has emerged. GAC focuses on reducing the environmental footprint of analytical processes by promoting the use of safer chemicals, minimizing waste, conserving energy, and improving method efficiency, without compromising analytical performance [1,2].

HPLC is widely used in food analysis due to its accuracy, sensitivity, and ability to detect and quantify a broad range of compounds, including nutrients, bioactive substances, contaminants, and residues, even at trace levels. Its broad analytical scope makes HPLC essential for quality control, authenticity testing, and ensuring compliance with food safety regulations [3,4]. Despite its analytical advantages, conventional HPLC methods often rely on hazardous organic solvents, such as acetonitrile and methanol, generate large volumes of chemical waste, and consume considerable amounts of energy through the operation of pumps, detectors, and column ovens. These environmental drawbacks are particularly significant in laboratories that process high sample loads on a routine basis. As a result, the long-term sustainability of HPLC is increasingly being questioned, prompting efforts to develop greener chromatographic practices with enhanced resource efficiency [5,6,7].

Promoting the application of green principles in HPLC has emerged as a strategic priority in analytical chemistry, driven by the need to reduce the environmental impact of chromatographic practices. GAC encourages the use of alternative solvents, reduction of reagent and sample volumes, energy-efficient instrumentation, and environmentally friendly sample preparation techniques [8,9,10]. In this context, innovation plays a central role, driving the evolution of greener instrumentation, miniaturized systems, and integrated workflows. Recent innovations, such as micro-HPLC, hybrid extraction systems, and solvent-free workflows, have demonstrated the feasibility of aligning HPLC with GAC principles in both research and industrial settings [11]. To support the implementation of greener chromatographic practices, several greenness assessment tools have been introduced, including the Analytical Eco-Scale, the Green Analytical Procedure Index (GAPI), and the AGREE metric (Analytical GREEnness), each offering quantitative or semi-quantitative evaluations of the environmental performance of analytical methods [9,10].

The aim of this review is to critically examine recent advances and innovations in green HPLC methodologies, with a particular focus on their applications in food analysis. It discusses how the principles of green analytical chemistry are being incorporated into chromatographic workflows, solvent selection, instrumental configurations, and regulatory approaches. In addition, it evaluates how the implementation of green strategies contributes to more sustainable food quality control and environmental responsibility, particularly in alignment with international legislative requirements and certification schemes [12]. This review also highlights key methodological trends, technological developments, and greenness assessment tools that support the transition toward more environmentally friendly chromatographic practices. Ultimately, it seeks to facilitate the practical adoption of green HPLC solutions by synthesizing up-to-date scientific knowledge in a form accessible to researchers, practitioners, and industry stakeholders. The review primarily emphasizes literature from the last five years, while also including a limited number of earlier foundational studies essential for understanding green analytical chemistry and its application in HPLC.

## 2. Principles of Green Analytical Chemistry

Green analytical chemistry is structured around twelve guiding principles that aim to reduce the environmental and human health impacts of analytical procedures while ensuring scientific robustness. These principles, first proposed by Galuszka et al. [13], establish a structured approach to developing and assessing analytical methods with sustainability as a key consideration. A summary of these twelve principles is presented in Table 1. 

Unlike traditional analytical approaches, which prioritize precision and selectivity often at the expense of environmental considerations, GAC integrates sustainability from the early stages of method development. This proactive approach supports both analytical performance and environmental responsibility [14,15].

### 2.1. Greenness Assessment Tools in Analytical Chemistry

To evaluate the environmental performance of analytical procedures, several greenness assessment tools have been developed and widely adopted in recent years. The Analytical Eco-Scale provides a penalty-point-based system that quantifies the deviation from an ideal green method based on solvent toxicity, energy consumption, waste generation, and occupational hazards [16,17]. Its simplicity and semi-quantitative nature make it particularly suitable for routine food analysis. In a recent study on phthalate detection in edible oils, the Eco-Scale was instrumental in highlighting the environmental drawbacks of conventional sample preparation methods involving large solvent volumes, while favoring miniaturized and solvent-free alternatives [17].

GAPI offers a visual, semi-quantitative evaluation that considers the entire analytical workflow, from sample collection to final determination, represented through a color-coded pictogram [18,19]. Each segment of the pictogram reflects specific stages of the method, enabling users to quickly identify critical steps in terms of environmental impact. In the assessment of HPLC methods for paclitaxel, GAPI was effective in distinguishing between high- and low-impact procedures, emphasizing the role of solvent choice and detection mode as key differentiating factors [19]. Recent advances have extended this approach with the development of the Complex-GAPI tool, which incorporates pre-analytical procedures and provides a more comprehensive evaluation of greenness [20,21]. In addition to that, the Modified GAPI (MoGAPI) introduces a scoring system and a dedicated software for more precise and comparable assessments [22].

The more recent AGREE metric, introduced in 2020, integrates all 12 GAC principles into a holistic algorithm, providing a single-score evaluation supported by intuitive graphic output [9,23]. The AGREE chart assigns scores on a scale from 0 to 1, delivering a normalized assessment of key parameters, such as solvent toxicity, energy consumption, sample preparation complexity, and analytical throughput. The latter refers to a method’s capacity to process high sample volumes efficiently, a critical consideration for routine laboratory workflows that directly impacts both sustainability and operational feasibility. This comprehensive evaluation enables rapid benchmarking and method optimization, while ensuring alignment with the principles of green chemistry. As such, AGREE is increasingly recognized as a robust and user-friendly tool for assessing the greenness of analytical procedures in both research and industrial settings [23]. More recently, the AGREEprep tool was introduced to specifically evaluate the greenness of sample preparation, addressing this critical step through ten assessment criteria and dedicated open-source software [24]. Table 2 provides a comparative overview of the major greenness assessment tools in analytical chemistry, summarizing their main characteristics, output formats, and distinctive features to facilitate direct comparison across different approaches.

These tools are not only theoretical constructs but are increasingly applied to assess and compare analytical procedures across various sectors. A recent bibliometric review of more than 100 studies confirmed a growing trend in the application of these metrics to optimize methods in food safety, pharmaceutical analysis, and environmental monitoring [3].

Recently, a new complementary tool to greenness assessment metrics, the Blue Applicability Grade Index (BAGI), has been introduced to address the practical and operational aspects of analytical methods, in alignment with the emerging concept of method applicability evaluation, as introduced by the principles of white analytical chemistry (WAC) and its Red–Green–Blue (RGB) model [25]. WAC seeks to balance analytical performance, environmental sustainability, and practical applicability, illustrated by the RGB model where red corresponds to method performance, green to environmental aspects, and blue to applicability. A “white” method is therefore one that harmonizes all three dimensions. BAGI evaluates ten key attributes related to applicability, including analysis type, throughput, reagent availability, automation, and sample preparation, and provides both a numeric score and a visual “asteroid” pictogram. While tools like AGREE assess the environmental sustainability of a method, BAGI emphasizes its practical viability and usability in real-world settings, making it particularly relevant for routine food laboratories. Its adoption in recent case studies demonstrates its value in identifying both strong and weak aspects of analytical workflows, and it is expected to gain wide acceptance alongside existing green metrics.

### 2.2. Application of GAC in Food Analysis

In food laboratories, where high sample throughput is common, the implementation of GAC principles offers clear advantages. These include reduced consumption of solvents and energy, enhanced occupational safety for analysts, and improved alignment with the requirements of green certification schemes [26].

The principles and tools of green analytical chemistry are increasingly applied in food analysis, a field that has traditionally depended on methods requiring large amounts of solvents and high energy consumption. Numerous studies have demonstrated the integration of GAC into sample preparation and chromatographic workflows for food matrices, employing green solvents such as ethanol and water, and minimizing hazardous reagent use [17,26]. Techniques such as QuEChERS (quick, easy, cheap, effective, rugged, and safe) and solid phase microextraction (SPME) are being widely adopted for the extraction of residues and contaminants from complex food samples, offering high efficiency while reducing solvent consumption and waste generation [17,26]. Visual assessment tools like GAPI and AGREE have been employed to evaluate the greenness of these methods across multiple food categories, including edible oils, fruits, dairy, and cereals [3,19,20,21,22,23,24].

Recent studies demonstrate the value of combining green assessment tools with miniaturized and solvent-free techniques, such as stir-bar sorptive extraction (SBSE) and supercritical fluid extraction (SFE), to achieve both analytical reliability and environmental sustainability in food safety and quality control [26]. SBSE provides a rapid and solvent-minimized approach, while SFE has emerged as a sustainable extraction tool that replaces large volumes of organic solvents with supercritical carbon dioxide. Such advancements further demonstrate the relevance and growing importance of GAC in food laboratories, where high sample volumes and strict regulatory demands require greener, safer, and more cost-effective analytical solutions.

### 2.3. Integration with Quality by Design (QbD)

The role of QbD in GAC is also gaining recognition. QbD approaches enable the systematic optimization of chromatographic conditions and solvent compositions with respect to both performance and sustainability criteria. By integrating green metrics into method validation and lifecycle management, laboratories can ensure continuous improvement aligned with GAC goals [27]. Recent studies highlight the synergy between QbD and GAC in optimizing method parameters using tools like analytical target profile (ATP), risk assessment (e.g., Ishikawa diagrams), and design of experiments (DoE) to enhance method robustness while minimizing environmental impact. Case studies in pharmaceutical and food analysis demonstrate that combining QbD with green metrics, such as GAPI, AGREE, and Eco-Scale, yields methods that are both analytically efficient and environmentally sustainable [27]. Furthermore, international collaborations and regulatory agencies are increasingly encouraging the integration of GAC metrics into method development protocols, positioning them as essential tools for next-generation analytical chemistry [28].

## 3. Green Innovations in HPLC

### 3.1. Eco-Friendly Solvent Systems

#### 3.1.1. General Classes of Green Solvents

The substitution of conventional organic solvents such as acetonitrile, chloroform, and dichloromethane with greener alternatives is a fundamental approach in the development of environmentally sustainable chromatographic methods [13,29,30]. Conventional solvents, despite their proven effectiveness in separation and detection, are associated with significant environmental and human health hazards due to their high volatility, toxicity, and persistence in ecosystems [13]. To mitigate these impacts, extensive research has focused on the identification and validation of low-toxicity, biodegradable, and renewable solvents for use as mobile phase components or extraction media in HPLC applications. These solvents act by reducing the polarity mismatch with analytes, ensuring efficient solubilization and extraction while simultaneously lowering toxicity and environmental load.

Ethanol and water, either used individually or as binary mixtures, represent the most commonly adopted bio-based alternatives due to their minimal environmental footprint, suitable polarity, and widespread availability. Ethanol, in particular, has been successfully employed as a single solvent or co-solvent in reversed-phase HPLC for the separation of diverse analytes, including caffeine, catechins, and phenolic acids [31,32]. In dietary and plant-derived matrices, ethanol-based mobile phases provide sufficient selectivity and resolution while enhancing the overall safety profile of the analytical procedure [31].

Ethyl lactate, produced through the esterification of lactic acid, constitutes another promising green solvent, offering excellent solvating capacity, high biodegradability, and low vapor pressure. Its applicability has been demonstrated in food, pharmaceutical, and cosmetic analyses, especially for compounds with intermediate polarity [33,34]. Similarly, limonene, a terpene obtained from citrus peel waste, has been explored as a feasible alternative to aromatic hydrocarbons in normal-phase liquid chromatography, combining favorable elution behavior with low toxicity [29].

#### 3.1.2. Emerging Green Solvent Systems

Natural deep eutectic solvents (NADES) and ionic liquids (ILs) represent a new generation of green solvents that enable the development of solvent systems optimized for specific analytical needs. NADES are typically composed of naturally occurring components such as sugars, amino acids, and organic acids, forming eutectic mixtures with low melting points and adjustable polarity. These solvents are non-volatile, biodegradable, and often edible, making them highly attractive for food-related applications [15,35]. Recent research demonstrates their compatibility with reversed-phase liquid chromatography, particularly for polar compound extraction from botanical and functional food matrices. Applications include the successful use of NADES for recovering phenolic compounds from fruit skins and peels, as well as flavor and aroma compounds from herbs and spices, highlighting their suitability across diverse food matrices [15]. Recent comprehensive studies highlight the increasing integration of NADES with sustainable extraction techniques such as ultrasound assisted extraction (UAE) and microwave-assisted extraction (MAE), demonstrating improved efficiency in recovering bioactive compounds from a wide range of food matrices and by-products while maintaining low toxicity and high biodegradability [10,36]. Such combined green approaches further support the practical application of NADES in food safety and quality analysis.

ILs, despite concerns regarding their synthesis and significant cost, offer a unique combination of low vapor pressure, high thermal stability, and adjustable solvation properties through careful modification of cation-anion pairs. They have been effectively integrated into sample preparation and HPLC workflows for the analysis of pharmaceutical residues, polyphenols, and endocrine-disrupting compounds in complex matrices [30,37]. When used appropriately and recycled effectively, ILs represent a viable green option in specialized contexts.

To support the systematic selection of green solvents, various assessment models have been developed. These include environmental impact metrics, hazard labeling systems, solvent selection guides grounded in the 12 Principles of green analytical chemistry, and other sustainability criteria [7,38,39]. These assessment tools help laboratories identify solvents that are not only environmentally preferable but also compatible with analytical performance requirements, availability, and regulatory compliance.

#### 3.1.3. Practical Considerations and Applications in Food Analysis

Among the most readily available green alternatives, aqueous ethanol and ethanol–water binary mixtures have been widely applied in chromatographic separations due to their low toxicity, biodegradability, and broad regulatory acceptance in food and pharmaceutical analyses [29,31,40,41]. Ethanol is particularly advantageous not only because of its favorable environmental profile but also due to its compatibility with reversed-phase stationary phases and common food matrix constituents. Various studies have confirmed the effectiveness of ethanol-based mobile phases for the separation of alkaloids, polyphenols, and flavor compounds in matrices, such as tea, chocolate, fruit extracts, and functional beverages [29,32,42].

In cases where analyte polarity or retention time adjustments are required, ternary systems incorporating ethanol, water, and a third green solvent, such as ethyl lactate or propylene carbonate, can be formulated to optimize separation efficiency while maintaining low environmental impact [33,34,43]. Ethyl lactate, a biodegradable solvent derived from lactic acid and ethanol, has shown high extraction efficacy for hydrophobic contaminants in oils and meat samples [34], while propylene carbonate has been explored as a feasible alternative to acetonitrile in routine food quality testing [33].

Furthermore, recent developments in solvent selection emphasize performance-based criteria, highlighting the importance of balancing environmental sustainability, analytical functionality, and chromatographic resolution. Metrics, such as the Analytical Eco-Scale and holistic scoring systems, enable the systematic comparison of solvent systems based on factors including energy consumption, waste generation, and toxicity profiles [13,38]. This approach supports the adoption of greener mobile phases without compromising method robustness, which is crucial for laboratories handling high sample volumes in food safety analysis [44].

### 3.2. Miniaturization and Micro-HPLC

Miniaturization within liquid chromatography (LC) has progressively become an essential strategy for integrating environmentally sustainable practices into analytical methodologies, in accordance with the principles of green analytical chemistry. In micro-HPLC, the use of narrow-bore columns and reduced flow rates enhances mass transfer and peak efficiency by decreasing longitudinal diffusion, thereby enabling faster separations with lower solvent consumption. By scaling down column dimensions and operating at lower flow rates, laboratories can achieve substantial reductions in solvent and sample consumption, while simultaneously enhancing detection sensitivity and separation performance [45,46,47,48]. This approach not only minimizes hazardous waste production but also supports cost efficiency and analytical sustainability across various applications, from food residue monitoring to omics research [49,50,51]. As technological advances in micro- and nano-HPLC instrumentation progress, the feasibility of widespread adoption in routine analysis continues to grow [52,53,54].

#### 3.2.1. Reduction in Solvent and Sample Volumes

One of the most practical benefits of LC miniaturization is the significant reduction in solvent use. Studies have revealed that transitioning from conventional analytical flow rates to micro-flow and nano-flow conditions can reduce solvent consumption by up to 95%, depending on the system configuration and application [45,46,48]. For example, micro-flow LC-MS/MS has been successfully applied in multiresidue pesticide analysis, achieving fivefold lower solvent use without compromising method sensitivity or reproducibility [47]. Similarly, micro-UHPLC-MS methods have shown a significant reduction in required sample volume while maintaining robust quantification of residues and contaminants in complex food matrices [46,52].

Reducing the internal diameter of chromatographic columns, from conventional 2.1 mm columns to capillary or micropillar formats, minimizes chromatographic dilution and consequently enhances ionization efficiency and detection sensitivity in tandem mass spectrometry [47,50,55]. However, the use of columns with reduced internal diameter inherently increases system backpressure, which may impose operational limitations and, in some cases, risk column failure if not properly managed [50,54,55]. Theoretical models suggest that reducing column inner diameter can concentrate analytes up to 49 times, translating to substantial sensitivity gains, especially for low-abundance targets in omics and single-cell analyses [55]. This scaling approach is consistent with the broader objective of developing greener and more resource-efficient analytical processes, while maintaining or even improving overall method performance [48,53].

#### 3.2.2. Use of Micro-Columns and Low-Flow Techniques

Recent advances in column design and instrumentation have significantly supported the practical implementation of miniaturized LC. Micro-columns, including micropillar array formats, provide controlled flow paths that minimize eddy dispersion and enhance separation efficiency, particularly under low-flow conditions [50]. These technological improvements enable efficient integration into existing instrumentation through the use of flow splitters or capillary detectors, thereby increasing the feasibility and cost-effectiveness of micro-LC applications [51]. In both environmental and food analysis, low-flow configurations such as nano-HPLC and capillary LC have demonstrated improved performance for complex matrices while markedly reducing solvent consumption [47,53,54].

Additionally, micro-flow techniques improve electrospray ionization (ESI) efficiency by producing smaller droplet sizes at the MS interface, resulting in enhanced ionization and signal-to-noise ratios [55]. Although factors such as dwell volume and extracolumn dispersion require careful control, advances in low-flow instrumentation and miniaturized components help address these challenges [54,55]. Emerging trends, including portable and lab-on-a-chip LC systems, further highlight the potential of miniaturization to enable on-site or in-situ analyses with reduced environmental impact [28,53,56]. For example, microfluidic LC devices have been successfully applied for rapid pesticide residue screening directly at production sites, significantly reducing sample transportation and solvent consumption [28,56].

#### 3.2.3. Operational Challenges and Perspectives

Although the advantages of miniaturized LC are well documented, its broader implementation continues to be constrained by technical and operational challenges. Requirements for specialized training, system maintenance, and higher initial investment may hinder its adoption in routine laboratory settings [56]. However, recent developments in modular instrumentation, microfluidic technologies, and standardized operating procedures are progressively mitigating these limitations [26,51]. In practice, several regulatory and food safety laboratories have already incorporated micro-flow and nano-HPLC systems for routine pesticide residue monitoring and testing for residues of pharmacologically active substances in various food matrices, including raw materials, end products, processed products, and foods of animal origin, highlighting the feasibility and versatility of such approaches [45,46,52]. As research and practice advance, miniaturization is increasingly recognized as a viable approach to improve both the environmental sustainability and analytical performance of contemporary HPLC methodologies [28,49,52]. Such applications demonstrate the potential of miniaturized LC techniques to support enhanced food safety compliance and sustainability objectives in the agri-food sector.

### 3.3. Sustainable Sample Preparation Prior to Chromatographic Analysis

The sample preparation stage is often the most material- and energy-demanding, as well as the most environmentally impactful step in analytical method development. In green analytical chemistry, the advancement and implementation of sustainable sample preparation techniques are critical for minimizing solvent usage, reducing hazardous waste generation, and enhancing the overall environmental performance of HPLC-based methodologies [57,58]. Recent reviews further emphasize that the integration of green chemistry principles into sample preparation is essential for improving food quality monitoring, ensuring consumer safety, supporting regulatory compliance, and promoting sustainability across the entire analytical process [59].

#### 3.3.1. Microextraction Techniques

Solvent-free or solvent-minimized techniques such as SPME, SBSE, fabric phase sorptive extraction (FPSE), and μ-solid phase extraction (μ-SPE) are gaining increasing attention due to their minimal environmental impact [60,61,62]. FPSE operates through the direct exposure of a sol–gel coated flexible substrate to the sample matrix, offering a large active surface area that enhances analyte sorption while reducing solvent consumption and sample handling steps. In μ-SPE, the reduced sorbent bed and smaller sample volumes enhance mass transfer efficiency, which accelerates extraction and decreases solvent consumption. These techniques are designed to reduce or eliminate the use of hazardous organic solvents and are typically based on equilibrium partitioning or adsorption mechanisms between the analyte and a sorbent phase. They use small quantities of materials, reduce sample treatment, and are compatible with direct coupling to HPLC systems, thereby minimizing sample losses and analytical variability.

SPME employs a polymer-coated fiber to extract analytes from liquid, solid, or gas phases and is particularly advantageous due to its simplicity, potential for automated operation, and minimal matrix interference. The principle of SPME relies on partitioning analytes between the sample matrix and the coated fiber, which minimizes the use of organic solvents and simplifies the workflow. It has been widely used for the extraction of volatile and semi-volatile compounds from fruits, juices, dairy products, and seafood [60].

FPSE utilizes flexible fabric substrates coated with sorptive materials, offering high extraction efficiency, short equilibration times, and strong mechanical stability, which is suitable for routine food testing [60]. Recent developments, such as magnet integrated FPSE (MI-FPSE), further enhance extraction efficiency by combining stirring and sorption mechanisms in a single device [63]. It has shown excellent performance in extracting pesticides and phenolic compounds from honey, olive oil, and herbal teas, and offers enhanced analyte recovery and broad applicability to various food matrices, including dairy, fruit-based beverages, and lipid-rich samples, without the need for extensive clean-up [61].

SBSE incorporates a magnetic stir bar coated with polydimethylsiloxane (PDMS) or other polymers to extract analytes during stirring, providing higher sorptive capacity compared to SPME. Applications include the analysis of aroma compounds and contaminants in wines, edible oils, and coffee [60,62].

μ-SPE involves miniaturized sorbent-packed devices and offers high preconcentration factors with low solvent volumes, making it ideal for trace-level analysis in complex matrices such as cereals, infant food, and meat products [60,62].

SBSE and SPME are frequently employed for the determination of contaminants and aroma compounds in complex food samples [60], especially for monitoring residues in products such as apples, milk, wine, and fish.

#### 3.3.2. Use of Green Solvents

Replacing traditional organic solvents, such as acetonitrile or dichloromethane, with greener alternatives is a key objective in sustainable sample preparation. These conventional solvents, although effective, are often volatile, toxic, and environmentally persistent [64]. In contrast, bio-based solvents like ethanol, ethyl lactate, and limonene are derived from renewable resources and exhibit low toxicity and high biodegradability, making them suitable for routine use in food laboratories [64].

Advanced solvent systems, including NADES and ILs, provide tunable physicochemical properties, such as adjustable polarity and hydrogen bonding capacity, enabling the selective extraction of a broad spectrum of analytes [65]. NADES, composed of natural metabolites like sugars, organic acids, and amino acids, are especially attractive due to their low cost, easy preparation, and compatibility with edible products. ILs, although less inherently green due to synthesis complexity, offer high thermal and chemical stability and are effective in the extraction of complex bioactive compounds from fatty or protein-rich matrices [65,66].

Supercritical carbon dioxide has emerged as an efficient, non-toxic, and residue-free extraction medium, particularly suitable for non-polar compounds [66]. Its adjustable solvating capacity, which can be finely modulated by controlling pressure and temperature conditions, enables the selective extraction of target analytes such as lipids, sterols, and essential oils in diverse food matrices [44]. Furthermore, its integration into sustainable extraction protocols has been extensively documented in the literature, particularly within agri-food systems and nutraceutical applications, where it demonstrates high extraction efficiency and environmental compatibility [44].

These green solvents are particularly suited for extracting both polar and non-polar compounds and have been applied successfully in the analysis of bioactive molecules (e.g., polyphenols, flavonoids, alkaloids) and food contaminants (e.g., pesticides, plasticizers, mycotoxins) in matrices such as fruits, vegetables, cereals, honey, and oils. This has been effectively demonstrated in the extraction of caffeine from coffee beans and aroma compounds from herbs and spices [44].

#### 3.3.3. Energy-Assisted Techniques

The integration of alternative energy sources into extraction processes can significantly improve extraction speed and reduce solvent and energy use. Techniques, such as UAE and MAE, are among the most established green approaches, as they enhance mass transfer and cell disruption, enabling shorter extraction times and lower solvent volumes [67]. These methods have shown excellent performance in extracting antioxidants, caffeine, and polyphenols from various plant-based foods and beverages, including tea, cocoa, and fruit peels [29,67].

Additionally, pressurized liquid extraction (PLE) and subcritical water extraction (SWE) represent promising green alternatives, especially for thermally stable analytes. These techniques utilize elevated temperatures and pressures to improve solubility and diffusion without the need for toxic solvents. When combined with bio-based solvents or used in solvent-free formats, they offer efficient extraction of polar compounds such as organic acids, flavonoids, and sugars [44,57]. Their application in matrices, like herbs, grains, and dried fruits, further demonstrates their suitability for sustainable food analysis, as highlighted in recent comprehensive reviews on green extraction trends in food chemistry [44]. Recent comprehensive reviews confirm that energy-assisted extraction techniques, including UAE, MAE, SWE, and SFE, play an important role in advancing sustainable and high-efficiency sample preparation in complex food matrices [68].

#### 3.3.4. Miniaturization and Integration Techniques

The miniaturization of sample preparation not only reduces the volume of solvents and samples needed but also enables faster and more efficient analytical procedures. By scaling down extraction formats, modern techniques achieve lower environmental impact without compromising analytical performance. Recent trends focus on the development of hybrid techniques, such as the combination of FPSE with QuEChERS, which integrates extraction and clean-up into a single streamlined step [60]. These integrated approaches are especially useful in food analysis, where complex matrices like spices, dairy products, and processed foods demand efficient sample handling with minimal matrix interference [60].

Moreover, miniaturized techniques contribute to improved reproducibility, lower method variability, and reduced operator exposure to harmful reagents and solvents [62]. Innovations in device design, such as portable μ-SPE cartridges and microfluidic-based extraction units, are further expanding the applicability of miniaturized techniques in routine food safety monitoring [62].

#### 3.3.5. Sustainability Assessment Tools

To objectively assess the environmental performance of green sample preparation techniques, evaluation tools such as GAPI, AGREE, and BAGI are increasingly employed [57]. These tools enable the comparative evaluation of analytical procedures based on parameters such as solvent use, energy consumption, sample volume, operational simplicity, and overall practical applicability, thereby facilitating continuous method optimization in alignment with the principles of green analytical chemistry [57].

In conclusion, green sample preparation techniques play a key role in improving the sustainability of chromatographic methods without compromising their analytical reliability. Their application is particularly critical in food analysis, where complex matrices, regulatory requirements, and the need for operational efficiency demand methods that are both effective and environmentally responsible. The integration of microextraction techniques, bio-based solvents, alternative energy sources, and structured greenness assessment tools enables the development of sample preparation protocols that meet performance requirements while advancing the goals of sustainable laboratory practice. Successfully scaling up these approaches from lab scale to industry scale remains a critical step to fully realize their potential in routine food quality control and safety assurance.

## 4. Applications in Food Analysis

### 4.1. Analysis of Bioactive Compounds

The accurate identification and quantification of bioactive compounds play a crucial role in the development and validation of functional foods and nutraceuticals, ensuring scientific substantiation of health claims and regulatory approval [69,70]. Polyphenols, carotenoids, alkaloids, and other phytochemicals contribute significantly to the health-promoting properties of plant-based foods, while their systematic analysis supports quality control, product standardization, and compliance with food safety and labelling regulations [69]. The adoption of advanced green extraction and chromatographic techniques, including innovative solvents and miniaturized approaches, minimizes solvent consumption, reduces hazardous waste, and enhances the overall sustainability and efficiency of food analysis [58,66].

Polyphenols and flavonoids represent the largest class of bioactive compounds investigated with green analytical approaches, reflecting both their nutritional significance and the extensive efforts to optimize extraction sustainability [44,58,66]. Various food matrices, including tea, bee products, fruit peels, and diverse agri-food by-products, have been successfully explored as valuable sources of these compounds. For instance, greener solvents like dimethyl carbonate and ethanol have replaced traditional toxic solvents in the extraction of caffeine and polyphenols from tea, improving safety and reducing environmental hazards [29,35]. UAE and MAE are commonly employed for the efficient recovery of phenolic compounds from pomegranate marcs, citrus peels, potato peels [71,72], and edible sprouts, demonstrating high extraction yields and significantly lower solvent consumption [73,74]. QuEChERS and other miniaturized extraction protocols [60,72] are increasingly applied to complex matrices such as honey and propolis, providing rapid, cost-effective, and solvent-minimized analytical procedures [75]. Additionally, integrated biomass processing methods and circular bioeconomy strategies help turn agri-food residues into high-value sources of polyphenols and flavonoids, supporting the conversion of waste streams into valuable products and sustainability goals [76,77].

Carotenoids and related pigments are important bioactives with well-established antioxidant and health-promoting effects that contribute to the functional value of many plant-based foods. Sustainable extraction methods, such as supercritical CO_2_ extraction and the use of NADES systems, have proven highly effective for recovering carotenoids from a variety of matrices, including seaweed, nettle leaves, citrus by-products, and Moringa oleifera [78,79,80,81]. UAE combined with green solvents like aqueous ethanol has been successfully optimized for the simultaneous recovery of carotenoids and vitamin C from orange peels, significantly reducing the reliance on conventional organic solvents and enhancing extraction selectivity [80]. Moreover, the application of supercritical fluid chromatography (SFC) and advanced green HPLC methods enables precise separation, improved quantification, and reliable profiling of carotenoids with minimal solvent use and lower environmental impact [82]. SFC, in particular, is increasingly recognized as a sustainable chromatographic tool, as it uses carbon dioxide as a primary mobile phase, thereby reducing dependence on conventional organic solvents.

Beyond polyphenols and pigments, green analytical strategies are increasingly applied for the reliable determination of alkaloids and other specialty bioactive compounds, supporting both product safety and regulatory compliance. Tea alkaloids, such as caffeine and theobromine, are typically quantified using miniaturized HPLC systems and greener mobile phases, which reduce solvent consumption and analytical costs while maintaining high sensitivity and precision [29,47]. *Moringa oleifera*, recognized for its diverse alkaloid and polyphenol profile, is often analyzed using a combination of UAE, MAE, and HPLC methods to enable detailed profiling of its nutritional and therapeutic constituents with minimal environmental impact [72,83]. Advanced extraction selectivity techniques are also being developed and optimized to enhance the isolation and purification of specific target compounds from challenging matrices, including seaweed and other complex plant-based foods, demonstrating the potential for sustainable and efficient methods in modern bioactive compound analysis [79].

Accurate and reliable determination of bioactive compounds is essential to support scientifically valid functional claims, clean-label product development, and full compliance with increasingly strict food safety and labelling regulations. Recent reviews highlight that robust analytical protocols aligned with green chemistry principles [69,84] not only improve the environmental sustainability of food testing processes but also build consumer confidence and promote market acceptance of health-oriented and eco-friendly food products. Recent literature further underscores this trend by highlighting innovative sample preparation strategies, such as pressurized liquid extraction, supercritical fluid extraction, and the use of natural deep eutectic solvents, which offer efficient recovery of bioactive compounds from diverse food matrices while significantly reducing solvent consumption and environmental impact [85].

In summary, the implementation of advanced green extraction and chromatographic techniques offers clear advantages for the sustainable analysis of bioactive compounds in a wide range of food matrices. Their continued integration into routine analytical procedures strengthens food quality assurance, facilitates compliance with regulatory standards, and contributes directly to the innovation and commercialization of functional foods with well-substantiated health benefits.

### 4.2. Detection of Contaminants and Residues

The detection of chemical contaminants and residues in food products is fundamental to safeguarding food safety and public health. As global food production intensifies to meet the growing demands of an expanding population, the widespread use of agrochemicals, veterinary drugs, food-processing aids, and packaging materials has inevitably led to the presence of diverse pollutants throughout the food supply chain [86]. The main classes of chemical contaminants include pesticide residues, mycotoxins, acrylamide, and veterinary drug residues, all of which pose potential risks for consumers if not effectively monitored and controlled [87]. In this context, integrated multi-residue analytical methods have become essential, enabling the simultaneous detection and quantification of diverse classes of contaminants in complex food matrices with improved efficiency and sustainability. However, conventional liquid chromatography-based methods for contaminant determination often require large sample volumes, generate significant amounts of hazardous waste, and demand energy-intensive procedures, all of which contribute to a substantial environmental footprint [88].

In response, the adoption of green analytical chemistry principles in contaminant analysis has accelerated in recent years, with a particular emphasis on the miniaturization and automation of sample preparation and separation steps [44]. Innovative approaches such as micro- and nano-UHPLC-MS now enable high-sensitivity, multiresidue analysis while drastically reducing solvent consumption and sample sizes, in some cases by more than 95% compared to conventional methods [46]. The integration of eco-friendly extraction techniques, solvent-free or low-toxicity methods, and renewable sorbent materials further enhances the sustainability of food contaminant testing, aligning laboratory practices with environmental and human health objectives [52].

Moreover, evolving policies such as the European Green Deal and the Farm to Fork strategy, together with strict regulatory requirements and ISO standard demands, actively promote the shift towards greener analytical methods, ensuring that contaminant control remains robust yet environmentally responsible [89]. As consumer awareness grows, the transition to sustainable detection methods for chemical residues in food will be crucial not only for compliance with maximum residue limits but also for supporting a safer, greener food system.

#### 4.2.1. Mycotoxins

Mycotoxins are toxic secondary metabolites produced mainly by filamentous fungi, including species of Fusarium, Aspergillus, and Penicillium, which commonly colonize food crops during cultivation and storage [90]. These compounds, typically of low molecular weight, can contaminate a wide range of agricultural products such as cereals, nuts, spices, coffee, wine, and dried fruits, and often persist through common thermal processing methods such as pasteurization [91]. The main mycotoxins of regulatory concern include aflatoxins, fumonisins, trichothecenes (notably deoxynivalenol, T-2 and HT-2 toxins), patulin, zearalenone, and ochratoxin A, all of which are linked to acute and chronic toxic effects, including hepatotoxicity, nephrotoxicity, immunosuppression, and carcinogenicity [92].

Despite their significance, conventional methods for mycotoxin analysis, typically based on liquid chromatography coupled with fluorescence, UV, or mass spectrometric detection, have notable drawbacks [93]. These traditional approaches often demand large sample volumes and complex, multi-step extraction and clean-up procedures that rely heavily on hazardous organic solvents, resulting in substantial chemical waste and increased environmental impact [94]. In addition, the complex nature of many food matrices further complicates the detection of trace levels of multiple co-occurring mycotoxins, placing high demands on analytical selectivity and sensitivity [95].

To address the public health risks posed by mycotoxins, strict maximum residue limits (MRLs) have been established by authorities such as the European Food Safety Authority (EFSA), World Health Organization (WHO), and the Codex Alimentarius Commission (CAC), with harmonized standards for different food products and animal feeds [96]. Nonetheless, mycotoxin contamination remains a significant challenge in global trade, requiring effective monitoring [90].

To face these analytical challenges, greener and more efficient extraction methods have been developed. Recent research highlights a clear shift towards sustainable sample preparation strategies that significantly reduce solvent use, sample size, and analysis time without compromising analytical performance. Miniaturized sorbent-based microextraction techniques, such as FPSE and SPME, have emerged as promising alternatives to conventional protocols, offering significant reductions in solvent use, sample size, and analysis time without compromising performance [97]. Advanced sorbent materials, including graphene oxide, magnetic nanoparticles, and other nanomaterials, are increasingly applied to improve extraction efficiency and selectivity [97,98].

Current studies show how these approaches are used for specific mycotoxins. For example, FPSE methods reinforced with graphene-based sorbents have been successfully applied for the determination of ochratoxin A in wine and cereal products [95,99]. Similar FPSE protocols have been developed for zearalenone analysis in food samples [96]. Nanoparticle-assisted FPSE has demonstrated substantial improvements in the detection of aflatoxins [98]. For trichothecenes, UPLC-MS/MS combined with green sample preparation has achieved sensitive detection of T-2 and HT-2 toxins in cereals [94].

These advances show how green extraction and chromatographic techniques can improve mycotoxin monitoring while reducing environmental impact. This helps laboratories comply with strict MRLs and promotes more sustainable food safety practices.

#### 4.2.2. Acrylamide

Acrylamide is a low-molecular-weight organic compound primarily formed in carbohydrate-rich foods through the Maillard reaction, involving asparagine and reducing sugars during thermal processing above 120 °C, such as frying, baking, and roasting. Common food sources include potatoes, bread, cereals, coffee, and snack products. Acrylamide is classified by the International Agency for Research on Cancer (IARC) as a Group 2A probable human carcinogen, with additional neurotoxic and genotoxic effects. Regulatory bodies such as EFSA and WHO have established benchmark levels and reference doses to mitigate its presence in foods [100,101,102]. 

Conventional methods for acrylamide detection, such as LC-MS/MS, GC-MS, and derivatization, require complex sample preparation with high solvent use and long analysis times. Common cleanup techniques like solid phase extraction add to environmental impact and cost. Moreover, complex food matrices (e.g., potato chips, bread, biscuits) challenge method sensitivity and selectivity [103,104,105].

To overcome these limitations, green analytical chemistry principles have inspired the development of miniaturized, solvent-minimized, and eco-friendly extraction and detection techniques. Novel sorbent materials, including dummy molecularly imprinted polymers (DMIPs) synthesized via aqueous-phase, bio-based monomers, magnetic nanoparticles, and ionic liquids, have been integrated into microextraction workflows such as magnetic solid-phase extraction and FPSE. These methods reduce solvent use, enhance selectivity, and simplify sample preparation, while maintaining or improving analytical performance [106,107,108].

Recent studies demonstrate the application of these green approaches across various food matrices. For instance, DMIP-based MSPE has been effectively applied to detect acrylamide in biscuits with low limits of detection and high recoveries [106]. Ionic liquid-based ultrasound-assisted extraction coupled with liquid chromatography has been successfully used for acrylamide determination in date seeds [107]. Modified QuEChERS methods incorporating eco-friendly solvents enable rapid and sensitive analysis of acrylamide in potato chips and bread [109]. Additionally, green electrochemical sensors employing polymer-coated graphite electrodes provide rapid, in situ acrylamide detection with minimal sample preparation [106].

These improvements enable more effective and environmentally responsible acrylamide monitoring, supporting regulatory compliance and contributing to sustainable food safety management.

#### 4.2.3. Pesticides

Pesticide residues are among the most widespread chemical contaminants in food, especially in fruits, vegetables, and cereal-based products, posing significant risks to public health through cumulative dietary exposure if MRLs are exceeded [45]. The intensive use of agrochemicals in conventional agriculture has resulted in complex multi-class contamination matrices, often involving dozens or even hundreds of different active substances within a single sample [110]. This complexity makes monitoring challenging, as food matrices can be highly variable and contain pigments, fats, or other co-extracted compounds that interfere with detection [111]. Traditional methods for pesticide analysis, such as conventional QuEChERS extraction followed by LC-MS/MS or GC-MS/MS, while robust and sensitive, still rely heavily on large volumes of toxic organic solvents and energy-intensive sample preparation steps [112]. Consequently, these methods contribute to high laboratory waste generation and environmental impact, conflicting with the principles of sustainable food safety management [113].

To address these challenges, recent research has focused on developing greener sample preparation methods that reduce solvent use, waste generation, and energy demands without compromising analytical performance [114]. Miniaturized extraction techniques, such as micro-scale QuEChERS, FPSE, and µSPEed^®^, have gained attention for their ability to handle complex matrices with minimal solvent volumes [115]. For example, FPSE has been successfully applied for the analysis of pirimicarb and fenitrothion residues in herbal teas, demonstrating high recoveries with reduced environmental impact [116]. Similarly, µSPEed^®^ combined with HPLC-MS/MS enables efficient extraction and quantification of plant alkaloid contaminants in tea, offering an eco-friendly approach adaptable for pesticides [117]. The μSPEed^®^ technique (a commercial variant of μ-SPE developed by ePrep) is based on the same principle of miniaturized solid-phase extraction, but employs specialized cartridges that enable faster extraction and lower solvent consumption. Sustainable adsorbents, including poly(ionic liquids), polydopamine-based materials, and deep eutectic solvent-modified supports, are emerging as promising sorbents for multi-residue pesticide pre-concentration, providing high selectivity and lower limits of detection in cereals, fruits, and vegetables [118]. Comparative studies in baby foods show that optimized green QuEChERS methods with novel cleanup sorbents can achieve regulatory compliance for pyrethrins and pyrethroids while significantly reducing solvent consumption [111]. Additional studies on minor crops have demonstrated the applicability of both GC and UPLC methods combined with greener extraction techniques to reliably monitor diverse pesticide residues [119]. These innovations demonstrate how green extraction strategies can support effective pesticide residue monitoring in line with the principles of green analytical chemistry.

In addition to greener extraction techniques, advances in detection technologies play a crucial role in sustainable pesticide residue analysis. High-resolution mass spectrometry (HRMS) and hybrid techniques like SFC-MS/MS have emerged as powerful tools for multi-residue screening with enhanced sensitivity and selectivity [120]. SFC-MS/MS, for instance, uses carbon dioxide as a green mobile phase, drastically reducing organic solvent consumption compared to conventional LC-MS/MS methods [120].

Moreover, untargeted and retrospective screening approaches allow laboratories to detect unexpected or emerging pesticide residues in complex food matrices, further strengthening consumer protection while optimizing resources [112]. Recent studies demonstrate how combining miniaturized QuEChERS or microextraction with HRMS enables the simultaneous detection of numerous pesticide residues in fruits, vegetables, and cereal products, with detection limits well below EU MRLs [45,112]. These integrated approaches demonstrate the synergy between green sample preparation and advanced detection, enabling practical routine monitoring in line with international standards.

The combination of greener extraction techniques with advanced detection strategies, laboratories can achieve reliable multi-residue pesticide monitoring that aligns with strict regulatory requirements, including EFSA maximum residue limits and the EU Directorate-General for Health and Food Safety (SANTE) guidelines [45,110,112]. These sustainable analytical approaches directly support the broader goals of the European Green Deal and the Farm to Fork strategy, which emphasize reducing the environmental impact of food production and ensuring safer, more transparent food supply chains [113]. As consumer demand for low-residue and sustainably certified products increases, the adoption of green analytical methods for pesticide detection will be essential to ensure regulatory compliance and to promote more environmentally responsible practices across the agri-food sector.

#### 4.2.4. Drug Residues

Veterinary drug residues are persistent chemical contaminants in animal-derived foods such as milk, meat, and fish and represent a public health concern due to antimicrobial resistance and toxicity when residue levels exceed MRLs [121]. Common residues include sulfonamides, penicillins, amphenicols, and tetracyclines like oxytetracycline and chloramphenicol, which require sensitive monitoring due to strict regulations [121,122].

Conventional extraction and detection methods are robust and well-established, but depend on large volumes of toxic solvents and intensive clean-up steps, generating significant waste. Recent advances, such as the use of ionic liquid-based dispersive liquid–liquid microextraction (IL–DLLME), demonstrate that greener sample preparation techniques can maintain high analytical performance while significantly reducing solvent consumption and environmental impact [123]. The method has been validated with multiple GAC assessment tools, confirming its suitability as a sustainable approach for residue monitoring in line with green analytical chemistry principles.

Green sample preparation techniques play a crucial role in minimizing the environmental footprint of veterinary drug residue analysis. Recent studies highlight greener alternatives such as FPSE, which has been applied for sulfonamides, amphenicols, and penicillins in milk, eliminating steps like protein precipitation and solvent evaporation while maintaining high recoveries [124,125,126]. Thus, FPSE has become a promising routine quality control practice in line with green analytical chemistry principles [125].

Other innovative approaches include the use of natural deep eutectic solvents or ILs for extraction, offering lower toxicity and improved selectivity for polar drug residues [123,127]. Miniaturized QuEChERS variants and microextraction techniques further reduce solvent and energy demands while handling complex food matrices such as milk, meat, and fish [122,127]. In summary, these developments confirm that greener extraction methods can strengthen routine residue control in line with sustainability goals.

Green chromatography plays an important role in advancing sustainable veterinary drug residue analysis. Traditional HPLC methods, although robust, often depend on large volumes of toxic organic solvents such as acetonitrile or methanol, contributing to high laboratory waste and environmental impact [123]. Recent studies demonstrate how greener approaches, including the use of ethanol or natural deep eutectic solvents as mobile phases, can significantly reduce solvent toxicity while maintaining separation efficiency for multi-class drug residues [123,128].

Validated green HPLC methods have been developed for sulfonamides, oxytetracycline, bromhexine, and flunixin in milk and beef, showing compliance with EU MRLs and excellent greenness scores according to established metrics such as the National Environmental Method Index (NEMI), Eco-Scale, and GAPI [128,129,130,131]. For example, the use of these assessment tools has confirmed that micellar liquid chromatography and UPLC-MS/MS methods can reliably estimate sulfadiazine and trimethoprim residues with significantly lower environmental impact [130]. Combined with miniaturized or solvent-free extraction steps, these greener chromatographic methods align residue monitoring with the principles of green analytical chemistry and support broader sustainability targets in food safety control. Recent applications further confirm this trend. For example, a novel UHPLC–MS/MS method based on molecularly imprinted solid-phase extraction was developed for the simultaneous determination of sulfonamide residues in fish, achieving high sensitivity and compliance with EU regulatory limits while minimizing solvent and reagent consumption [132]. This study highlights how advanced sorbent technologies, when combined with modern chromatographic platforms, can provide both robust residue monitoring and significant environmental benefits.

Τhe implementation of greener extraction and chromatographic strategies is vital for ensuring reliable veterinary drug residue monitoring with reduced environmental impact. The broader adoption of validated green methods and systematic greenness assessment will help strengthen compliance with international standards while supporting sustainability goals across the food supply chain.

### 4.3. Green Analytics for Clean-Label and Sustainability Claims

The increasing consumers’ demand for clean-label and eco-labeled food products has intensified the need for robust analytical approaches that can verify claims of natural origin, minimal processing, and sustainable sourcing. Certification schemes, such as ISO 14024 (Type I environmental labeling), complement regulatory requirements by providing transparent eco-labelling criteria and third-party verification [133]. The EU Ecolabel, for example, operates under these standards and demands independent third-party verification, highlighting the critical role of reliable analytical data to substantiate environmental performance [134].

However, recent reviews have pointed out that while eco-labels have clear benefits in guiding sustainable choices, they may face limitations related to consumer comprehension, trust, and inconsistent application when not properly supported by transparent and standardized testing [135,136]. Younger consumers are shown to value sustainability but expect greater clarity and traceability, demonstrating the need for trustworthy green analytical practices that verify environmental claims [137].

Green analytical chemistry contributes directly to this process by replacing hazardous solvents with safer alternatives, miniaturizing extraction and separation steps, and reducing the overall environmental impact of test methods [38,44]. For example, clean-label extraction of bioactive compounds from food waste through MAE demonstrates how greener techniques can add value while supporting sustainability narratives [8]. Similarly, adopting greener separation methods and clear criteria for evaluating the environmental impact of analytical processes can enhance the credibility of clean-label and eco-label claims in the market [28,38].

Recent advances in sustainable food profiling have shown that green analytical strategies can be effectively applied to authentication processes, thereby strengthening traceability and enhancing the credibility of eco-label and clean-label claims [138]. Reliable, reproducible, and independently verified data are essential to prevent misleading claims and to ensure that eco-labels deliver real environmental benefits [133,135,136]. This highlights the gap between consumer expectations and the analytical capacity to verify such claims, especially as supply chains become more complex. In practice, this means that food producers need to integrate validated green methods into routine quality control to align marketing claims with measurable sustainability outcomes. Overall, integrating advanced green analytical methods is not only essential for regulatory compliance but also for reinforcing consumer confidence in the authenticity of sustainability and clean-label statements [135,137,139,140,141]. Continued innovation in green analytical chemistry will be essential to support the credibility and wider adoption of clean-label and eco-label initiatives. To enhance clarity and provide a concise overview, Table 3 summarizes the main applications of green HPLC in food analysis, linking application areas, applied green approaches, and representative food matrices.

## 5. Regulatory Compliance and Certification Schemes

### 5.1. ISO 14001 and Green Laboratory Practices

The ISO 14001 standard provides a globally recognized structure for implementing Environmental Management Systems (EMS) in organizations, including analytical laboratories. Its application in laboratory environments supports systematic improvement in environmental performance, compliance with regulatory requirements, and responsible resource management [142]. The standard encourages laboratories to monitor and control their environmental aspects, such as chemical usage, waste generation, and energy consumption, through a structured cycle of planning, implementation, evaluation, and continuous improvement.

Green analytical chemistry complements ISO 14001 by offering practical strategies for reducing the environmental footprint of analytical activities. Techniques that minimize solvent use, reduce hazardous waste, and improve energy efficiency are directly aligned with ISO 14001 objectives. Moreover, green metrics and method selection criteria rooted in GAC provide quantifiable indicators that can be integrated into EMS documentation and performance evaluations [143].

Recent studies have shown that laboratories adopting ISO 14001 benefit from enhanced environmental awareness, structured waste handling, and more sustainable laboratory workflows. The integration of GAC-aligned practices, such as the use of safer solvents and energy-efficient instrumentation, facilitates compliance with ISO 14001 and contributes to improved operational sustainability. Recent evaluations in clinical and analytical laboratory environments have shown clear improvements in environmental performance, including lower energy consumption, reduced chemical waste, and decreased operating costs [144]. Moreover, the environmental indicators emphasized in ISO 14001, such as solvent use, energy efficiency, and waste generation, are closely aligned with the evaluation criteria of green analytical practices, enabling coherent integration into EMS performance monitoring.

### 5.2. Integration of ISO 22000 and HACCP

Green HPLC practices are increasingly compatible with food safety management systems such as ISO 22000, which emphasize risk-based thinking, process integration, and continuous improvement through the plan-do-check-act (PDCA) cycle [145]. The standard has been built upon HACCP principles by organizing food safety protocols around preventive controls and verification mechanisms, which serve as effective entry points for incorporating green analytical tools [146].

Under ISO 22000, analytical procedures are integrated into prerequisite programs (PRPs), operational controls, and validation steps. The use of environmentally conscious chromatographic methods, such as those employing low-toxicity solvents or miniaturized formats, can enhance the effectiveness of these programs while also reducing environmental impact. The alignment of green analytical chemistry with ISO 22000 objectives supports both regulatory compliance and sustainable innovation [147].

A recent study on food facilities with multiple production lines demonstrated that the implementation of ISO 22000:2018 not only reduced non-compliance incidents by over 40%, but also improved operational indicators such as downtime and traceability [148]. The study highlighted the importance of analytical improvements, particularly in hazard analysis and monitoring, in achieving these outcomes. Integrating green HPLC into the verification of critical control points (CCPs) and supporting decision-making through reliable, low-impact analysis strengthens the connection between food safety and environmental performance.

### 5.3. ESG and GFSI Alignment

Environmental, social, and governance (ESG) criteria are increasingly influencing the strategic orientation of stakeholders in the food industry, including analytical laboratories. ESG-related policies and performance indicators encourage the adoption of environmentally responsible practices such as energy efficiency, solvent reduction, and waste minimization. Within this context, GAC provides a practical approach to enhancing the environmental pillar of ESG performance, particularly in laboratories engaged in food safety and quality analysis. Recent literature highlights the growing significance of ESG disclosure in the agri-food sector and its alignment with broader sustainable development goals [149].

Given the rising importance of ESG criteria across sectors, the Global Food Safety Initiative (GFSI) has emerged as a key driver in the global harmonization of food safety standards. Since its establishment in 2000, GFSI has developed into a comprehensive multi-stakeholder platform that benchmarks food safety certification programs, facilitates regulatory alignment, minimizes audit redundancy, and fosters trust across the food supply chain [150]. Contemporary GFSI-recognized certification schemes, including FSSC 22000, IFS, and BRC, increasingly incorporate environmental and sustainability considerations into their updated audit protocols, thereby expanding the scope of food safety systems to align with broader ESG objectives [150,151].

Surveys of certified food processors reveal broad support for GFSI’s contribution to enhanced food safety practices, streamlined third-party auditing processes, and improvements in documentation and personnel training [152]. Moreover, the integration of GAC practices, such as solvent-free extraction techniques and energy-efficient instrumentation, within GFSI-certified operations can reinforce both food safety outcomes and ESG reporting effectiveness. This alignment contributes to the advancement of sustainable and transparent food systems, in line with the objectives promoted by the GFSI and its strategic focus on cultivating a strong food safety culture [153].

Furthermore, recent systematic reviews of ESG disclosure literature provide deeper insight into the theoretical foundations and emerging themes that guide ESG practices across industries, including the food sector. ESG disclosure is increasingly influenced by regulatory requirements, stakeholder expectations, and corporate governance mechanisms, all of which are directly relevant to food businesses aiming to align with global sustainability goals [154]. Additionally, ESG performance is influenced by a range of interrelated factors that increasingly intersect with food safety and analytical practices. These include stakeholder pressure, internal organizational capabilities, corporate governance structures, institutional context, and regulatory requirements [155]. Such factors influence how organizations incorporate ESG strategies into operational decision-making processes, including quality assurance and sustainability reporting. In the context of food sector certification, the alignment of GFSI practices with ESG drivers underscores the importance of adopting greener analytical workflows, not only to ensure compliance but also to promote environmental responsibility and strengthen market credibility. To provide an integrative overview, Table 4 summarizes the main green HPLC approaches in relation to their applications in food analysis and their alignment with regulation, certification, and sustainability.

As shown in Table 4, green HPLC approaches not only enhance analytical performance and environmental sustainability but also align directly with food safety regulations, certification schemes, and broader sustainability goals. This integrated perspective underlines the role of green HPLC as both a scientific and regulatory tool in sustainable food analysis.

## 6. Challenges and Future Perspectives

Although sustainable practices are increasingly being prioritized in analytical chemistry, the widespread adoption of green HPLC still faces multidimensional challenges. One of the primary limitations is the high initial cost of eco-friendly instrumentation and specialized equipment, which may deter small and medium-sized laboratories from transitioning to greener alternatives. Additionally, concerns regarding analytical performance, such as sensitivity or reproducibility when using greener solvents or miniaturized techniques, continue to limit full integration into routine workflows. The limited availability of validated green methodologies and standardized protocols further complicates adoption, particularly in regulated sectors such as food analysis.

The need for targeted training and capacity building is also critical, as laboratory staff must develop the skills required to implement and maintain advanced green HPLC techniques and equipment. Moreover, international cooperation and the sharing of best practices can help accelerate the development and harmonization of green HPLC protocols, particularly in regions where access to advanced technologies remains limited.

However, significant opportunities lie ahead. Automation and digitalization are rapidly transforming analytical laboratories, offering the potential to optimize processes, reduce resource consumption, and support real-time data monitoring for better decision-making. The integration of smart technologies can further enhance the sustainability and efficiency of HPLC systems. Recent studies demonstrate that NADES-assisted extraction combined with HPLC and machine learning can deliver highly efficient, automated solutions for complex analyte determination, while AI-driven method development can simultaneously optimize chromatographic performance and greenness [156,157]. These advances align with the broader concept of smart analytical chemistry—integrating the principles of green, sustainable, and white analytical chemistry with emerging technologies and miniaturized platforms—to accelerate innovation in food analysis [158]. In parallel, recent innovations in green analytical chemistry emphasize balancing analytical performance with environmental sustainability, including the incorporation of tools such as life cycle assessment to systematically evaluate the ecological footprint of chromatographic methodologies [5]. Together, these perspectives indicate that the future development of green HPLC will be driven by digital transformation, systemic sustainability assessments, and interdisciplinary collaboration to ensure both analytical robustness and measurable reductions in environmental impact. Looking forward, there is strong potential for the formal integration of green analytical chemistry principles into international food safety and quality standards. By embedding sustainability criteria into regulatory requirements and certification schemes, stakeholders across the food supply chain can be encouraged to adopt greener analytical practices as part of their compliance obligations. Collaborative efforts among scientists, regulators, and industry stakeholders will be essential to ensure that advancements in green HPLC methodologies translate into improvements in analytical performance and measurable reductions in environmental impact.

## 7. Conclusions

Green innovations in HPLC represent a meaningful advancement toward the implementation of more sustainable analytical practices in food analysis. The use of low-toxicity and sustainable solvents, miniaturized system configurations, and energy-efficient instrumentation has demonstrated that reductions in the ecological footprint of chromatographic workflows are achievable without compromising analytical robustness. Moreover, the incorporation of green sample preparation techniques further enhances the sustainability of HPLC applications, particularly in the analysis of complex food matrices.

This review demonstrates that green HPLC is not only feasible but also effective, delivering measurable benefits for laboratories, the environment, and the broader food sector. As awareness increases and regulatory requirements continue to evolve, the broader implementation of green analytical strategies becomes both relevant and necessary. Integrating such approaches into food safety and quality control systems can contribute to the achievement of sustainability objectives, while also promoting methodological innovation and enhancing the resilience of analytical operations.

Ultimately, the advancement of green analytical chemistry in food analysis requires interdisciplinary collaboration. Coordinated efforts among analytical chemists, food scientists, instrument manufacturers, and regulatory bodies are essential for the development and implementation of robust, environmentally responsible methodologies. To support broader adoption, actions such as targeted training, method harmonization and standardization, and the inclusion of green criteria in regulatory requirements and certification schemes are also necessary. Strengthening these systemic supports will position green HPLC as an integral component of routine food safety and quality control, reinforcing both sustainability commitments and analytical excellence.

## Figures and Tables

**Table 1:** The twelve principles of green analytical chemistry (GAC), summarizing key strategies for minimizing the environmental impact of analytical methods.

No.	Principle	Description
1	Direct techniques	Use direct analytical techniques to minimize extensive sample preparation.
2	Reduced sample size	Reduce sample size and number of samples to limit material consumption and waste.
3	In situ measurements	Favor in situ measurements to avoid transport and contamination risks.
4	Waste minimization	Minimize waste generation at every stage of the analytical process.
5	Safer solvents/reagents	Select safer solvents and reagents to reduce toxicity.
6	Avoid derivatization	Avoid derivatization to limit chemical use and waste.
7	Energy efficiency	Minimize energy consumption through energy-efficient instrumentation and conditions.
8	Miniaturization/reagent-free	Develop reagent-free or miniaturized methods.
9	Automation/integration	Use automation and integration to enhance efficiency and reduce errors.
10	Multi-analyte approach	Adopt multi-analyte or multi-parameter methods.
11	Real-time analysis	Pursue real-time analysis for timely decision-making and waste avoidance.
12	Greenness assessment	Apply greenness metrics to quantify and improve environmental performance.

**Table 2:** Key characteristics and schematic representation of the principal greenness assessment tools applied in analytical chemistry, including their main focus, output type, and notable features.

Tool	Graphical Representation	Main Focus	Output Type	Notable Features	Ref.
GAPI	** 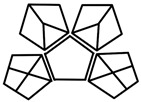 **	Entire analytical workflow	Color-coded pictogram	Easy visualization, no total score	[17]
BAGI	** 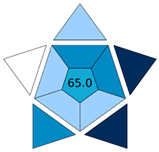 **	Workflow + total score	Pictogram + % score	Integrates Eco-Scale scoring	[18]
Complex-GAPI	** 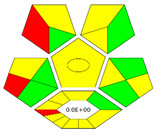 **	Includes pre-analytical steps	Extended pictogram	More comprehensive greenness coverage	[19]
AGREE	** 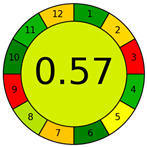 **	12 principles of GAC	Radial chart (0–1)	Holistic single-score metric	[20]
AGREEprep	** 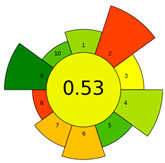 **	Sample preparation	Pictogram + score	First dedicated sample prep metric	[21]

**Table 3:** Summary of green HPLC applications in food analysis.

Application Area	Green Approaches Applied	Example Food Matrices
Bioactives (polyphenols, carotenoids, alkaloids)	Green solvents (EtOH, NADES), UAE/MAE, SFC, micro-HPLC	Tea, citrus peels, pomegranate, seaweed, bee products, agri-food by-products
Contaminants and residues (pesticides, mycotoxins, acrylamide, veterinary drugs)	Miniaturized QuEChERS, FPSE, IL-DLLME, green HPLC phases	Cereal products, honey, potato chips, milk, fish, fruits, and vegetables
Clean-label and sustainability claims	Eco-friendly extraction, green profiling, chemometrics	Honey, plant-based foods, eco-labelled products

**Table 4:** Link between green HPLC approaches, applications in food analysis, and regulation, certification, and sustainability.

Green HPLC Approach	Application in Food Analysis	Regulation, Certification, and Sustainability
Eco-friendly solvents	Determination of bioactive compounds (polyphenols, vitamins, natural pigments)	ISO 14001 (environmental management)
Miniaturization (µ-HPLC)	Detection of contaminants and residues (pesticides, veterinary drugs)	ISO 22000/HACCP (food safety)
Energy-efficient methodologies	Clean-label and sustainability claims (additives, processing markers)	ESG criteria, GFSI alignment
Green sample preparation	Broad applicability across food matrices	Contribution to sustainable consumption and production

## Abbreviations

The following abbreviations are used in this manuscript:

HPLC	high-performance liquid chromatography
GAC	green analytical chemistry
GAPI	Green Analytical Procedure Index
AGREE	analytical GREEnness
BAGI	Blue Applicability Grade Index
WAC	white analytical chemistry
RGB	red–green–blue
QuEChERS	quick, easy, cheap, effective, rugged, and safe
SPME	solid phase microextraction
SBSE	stir-bar sorptive extraction
SFE	supercritical fluid extraction
ATP	analytical target profile
QbD	quality by design
DoE	design of experiments
NADES	natural deep eutectic solvents
ILs	ionic liquids
UAE	ultrasound assisted extraction
MAE	microwave-assisted extraction
ESI	electrospray ionization
PDMS	polydimethylsiloxane
MI-FPSE	magnet integrated FPSE
PLE	pressurized liquid extraction
SWE	subcritical water extraction
SFC	supercritical fluid chromatography
MRLs	maximum residue limits
EFSA	European Food Safety Authority
WHO	World Health Organization
CAC	Codex Alimentarius Commission
IARC	International Agency for Research on Cancer
DMIPs	dummy molecularly imprinted polymers
HRMS	high-resolution mass spectrometry
SANTE	EU Directorate-General for Health and Food Safety guidelines
NEMI	National Environmental Method Index
EMS	Environmental Management Systems
PDCA	plan-do-check-act
PRPs	prerequisite programs
CCPs	critical control points
ESG	environmental, social, and governance
GFSI	Global Food Safety Initiative
